# Association of Ibuprofen Prescription With Acute Kidney Injury Among Hospitalized Children in China

**DOI:** 10.1001/jamanetworkopen.2021.0775

**Published:** 2021-03-04

**Authors:** Licong Su, Yanqin Li, Ruqi Xu, Fan Luo, Qi Gao, Ruixuan Chen, Yue Cao, Sheng Nie, Xin Xu

**Affiliations:** 1 Division of Nephrology, National Clinical Research Center for Kidney Disease, State Key Laboratory of Organ Failure Research, Nanfang Hospital, Southern Medical University, Guangzhou, China

## Abstract

**Importance:**

Ibuprofen is widely used in children worldwide, especially in those with cancer, fever, or trauma. However, large and high-quality studies of the association between ibuprofen and acute kidney injury (AKI) in children have been lacking.

**Objective:**

To examine the association between the use of ibuprofen and the risk of hospital-acquired AKI in hospitalized children in China.

**Design, Setting, and Participants:**

This cohort study analyzed the cohort of the Epidemiology of AKI in Chinese Hospitalized Patients (EACH) study, a large, multicenter retrospective study of 3 044 023 patients who were admitted to 1 of 25 academic medical centers in China between January 1, 2013, and December 31, 2015. Patient-level data were obtained from the electronic health record system of the participating centers. Hospitalized children aged 1 month to 18 years who had prescriptions and a certain number of serum creatinine (SCr) tests were included. Children with end-stage renal disease, community-acquired AKI, low baseline SCr level (<10 μmol/L), high standardized baseline SCr level (>4 times the sex- and age-specific reference value), or missing diagnosis code were excluded. Data analysis was conducted from January 1, 2020, to August 30, 2020.

**Exposures:**

Exposure to ibuprofen was coded as a time-dependent dichotomous variable.

**Main Outcomes and Measures:**

Baseline SCr level was calculated for each patient as the mean of all available SCr values between the 30 days prior to admission and the first SCr testing within the first 3 days of hospitalization. Acute kidney injury was defined as an increase in SCr level of 26.5 μmol/L or higher within 48 hours or by 50% or more over the baseline value, according to the Kidney Disease: Improving Global Outcomes guidelines.

**Results:**

Among the 50 420 children (mean [SD] age, 5.0 [5.2] years; 30 640 boys [60.8%]) included in this study, 5526 (11.0%) used ibuprofen and 3476 (6.9%) developed hospital-acquired AKI during hospitalization. Ibuprofen use was associated with a statistically significantly increased risk of hospital-acquired AKI (hazard ratio [HR], 1.23; 95% CI, 1.14-1.34) after adjusting for confounders. Ibuprofen use was associated with a greater hazard in children who had chronic kidney disease vs those without (HR, 2.31 [95% CI, 1.73-3.10] vs 1.19 [95% CI, 1.09-1.29]), required intensive care vs those without this need (HR, 1.47 [95% CI, 1.24-1.75] vs 1.18 [95% CI, 1.07-1.29]), or were older vs younger (>10 years and >1 year to 10 years vs 1 month to 1 year) (HR, 1.64 [95% CI, 1.32-2.05]; 1.36 [95% CI, 1.23-1.52] vs 0.99 [95% CI, 0.86-1.13]). Dose-response analysis suggested that the association of ibuprofen with the risk of hospital-acquired AKI was dose-dependent.

**Conclusions and Relevance:**

This study found that ibuprofen was widely used and associated with an increased risk of hospital-acquired AKI in hospitalized children in China. The judicious use of ibuprofen and close monitoring of kidney function in children are needed.

## Key Points

**Question:**

What is the association of prescribed ibuprofen with the risk of hospital-acquired acute kidney injury among hospitalized children?

**Findings:**

In this cohort study of 50 420 hospitalized children in China, ibuprofen use was associated with an increased risk of hospital-acquired acute kidney injury, and the effect size appeared to be dose-dependent.

**Meaning:**

Results of this study suggest that pediatricians should be aware of the potentially increased risk of acute kidney injury associated with a high dose of ibuprofen among hospitalized children; judicious use of ibuprofen and close monitoring of kidney function are necessary.

## Teaser

This cohort study evaluates the risks and incidence of acute kidney injury observed in hospitalized children who received ibuprofen, the most commonly prescribed nonsteroidal anti-inflammatory drug in the world.

## Introduction

Acute kidney injury (AKI) is an abrupt decline of kidney function that occurs over hours or days. It is a common complication among hospitalized patients^1^ and is associated with a substantially increased risk of death, a longer length of stay (LOS), and other adverse outcomes.^2,3,4,5,6^ It has become a public health problem worldwide, with incidences ranging from 11.6% to 18.3% in adults and from 19.6% to 26.9% in children.^2,3,7,8,9,10^ Exposure to medications with nephrotoxic effects is a common cause of hospital-acquired AKI, accounting for a substantial portion of the total incidence of AKI in children, particularly critically ill children.^11,12^ Therefore, avoiding inappropriate use of drugs with nephrotoxic effects is an important strategy to prevent iatrogenic hospital-acquired AKI in children.

Ibuprofen, a nonsteroidal anti-inflammatory drug (NSAID), is a nonselective blocker of cyclooxygenase. It is frequently prescribed in children for diverse therapeutic indications, including fever, postoperative pain, tumors, and inflammatory disorders, such as juvenile idiopathic arthritis and Kawasaki disease. In the United States, an estimated 36 million people use nonprescription analgesics on a daily basis, and more than 111 million NSAID prescriptions are dispensed each year.^13^ Currently, more than 20 million prescriptions for ibuprofen are filled yearly in the US, and this number does not take into account its vast over-the-counter use.^14^ As a blocker of cyclooxygenase activity, ibuprofen inhibits the synthesis of prostaglandin, which leads to vasoconstriction, increased preglomerular resistance, decreased renal perfusion, and increased risk of prerenal AKI.^12,15^

Use of NSAIDs has been associated with an increased risk of AKI in both adults and children.^16,17,18^ A previous study showed that the use of NSAIDs was associated with a 63% increase in the risk of AKI and accounted for 11% of the total risk of hospital-acquired AKI among Chinese children.^3^

Although ibuprofen is the most commonly prescribed NSAID in the world, few large studies have specifically studied the association between ibuprofen and hospital-acquired AKI in children.^19,20,21,22,23,24,25^ Furthermore, these studies were generally limited by a small sample size and reported inconsistent findings. In this multicenter cohort study, we examined the association between the use of ibuprofen and the risk of hospital-acquired AKI in a large, retrospective cohort of hospitalized children in China.

## Methods

The Medical Ethics Committee of Nanfang Hospital, Southern Medical University approved the study protocol and waived patient informed consent because this study was retrospective. We followed the Strengthening the Reporting of Observational Studies in Epidemiology (STROBE) reporting guideline.^26^

### Study Design, Population, and Data Source

The study population was drawn from the cohort of the Epidemiology of AKI in Chinese Hospitalized Patients (EACH) study, a large, multicenter retrospective study of 3 044 023 patients who were admitted to 1 of 25 third-tier medical centers in China between January 1, 2013, and December 31, 2015. Each of the 25 participating centers (Children’s Hospital of Nanjing Medical University, Nanjing; Children Hospital of Zhejiang University, Hangzhou; Anhui Institute of Pediatric Research, Anhui Provincial Children’s Hospital, Hefei; Guangzhou Women and Children’s Medical Center, Guangzhou Medical University, Guangzhou; Children’s Hospital of Fudan University, Shanghai; First Affiliated Hospital of Zhengzhou University, Zhengzhou; Chengdu Women and Children’s Central Hospital, Chengdu; Shanghai Children’s Medical Center, Shanghai Jiaotong University, Shanghai; Jinan Children’s Hospital, Jinan; Pediatric Medical Research Center, Gansu Province Child’s Hospital, Lanzhou University Second Hospital, Lanzhou; West China Second University Hospital, Sichuan University, Chengdu; Sichuan Provincial People’s Hospital, University of Electronic Science and Technology of China, Chengdu; Guangdong General Hospital, Guangdong Academy of Medical Sciences, Guangzhou; Children’s Hospital of Chongqing Medical University, Chongqing; Guizhou Provincial People’s Hospital, Guizhou University, Guiyang; The Second Affiliated Hospital, Zhejiang University, Hangzhou; Guilin Medical University Affiliated Hospital, Guilin; Tongji Hospital Affiliated to Tongji Medical College, Huazhong University of Science and Technology, Wuhan; Kidney Disease Center, the First Affiliated Hospital, Zhejiang University, Hangzhou; Center for Nephrology and Urology Shenzhen University, the First Affiliated Hospital of Shenzhen University, Shenzhen University, Shenzhen; the Second Affiliated Hospital of Dalian Medical University, Dalian; Huashan Hospital, Fudan University, Shanghai; Institute of Nephrology, Zhong Da Hospital, Nanjing; and Sun Yat-sen Memorial Hospital, Sun Yat-sen University, Guangzhou) exported the hospitalization records as well as the laboratory and prescription data of all hospitalized patients within the study period from its proprietary electronic health record system.

The hospitalization records consisted of demographic characteristics such as age, sex, date of diagnosis, diagnosis code at admission and discharge, operation procedures and dates, need for intensive care, in-hospital death, and total hospitalization cost. The laboratory data included the results and time of serum creatinine (SCr) tests. The prescription data included names, doses, and start and stop times of the drugs or procedures prescribed.

The exported data from all participating centers were pooled and cleaned at the National Clinical Research Center for Kidney Disease in Guangzhou, China. All of the laboratories of the participating centers had passed the annual External Quality Assessment of the Chinese National Center for Clinical Laboratories.

**Figure 1.**
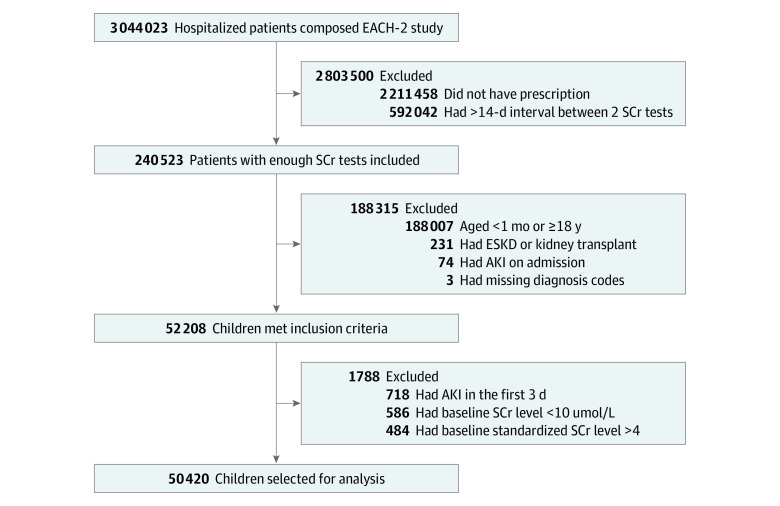
Flowchart of Patient Selection AKI indicates acute kidney injury; EACH, Epidemiology of AKI in Chinese Hospitalized Patients study; ESKD, end-stage kidney disease; and SCr, serum creatinine.

We included hospitalized children aged 1 month to 18 years who underwent SCr testing at least once during the first 3 days of hospitalization and at least 1 additional SCr test within 14 days of the initial SCr test. We excluded children with end-stage kidney disease (n = 231) or a missing diagnosis code (n = 3); children with a low baseline SCr level of less than 10 μmol/L (n = 586), which we considered to be unstable; and children with a high baseline SCr level of more than 4 times the sex- and age-specific reference value (n = 484), which indicates severe loss of kidney function. Patients with an *International Statistical Classification of Diseases, Tenth Revision, Clinical Modification* (*ICD-10-CM*) code of N17.051, N17.153, N17.252, N17.851, and O90.451 on admission or an AKI detected in the first 3 days of hospitalization were considered as having community-acquired AKI and were excluded (n = 792). A total of 50 420 children were selected for analysis (Figure 1).

### Hospital-Acquired AKI Identification, Drug Exposure, and Comorbidities and Clinical Procedures

A baseline SCr level was calculated for each patient as the mean of all available SCr values between the 30 days before admission and the first SCr testing within the first 3 days of hospitalization. Acute kidney injury was defined as an increase in SCr level of 26.5 μmol/L or higher within 48 hours or by 50% or more over the baseline value according to the Kidney Disease: Improving Global Outcomes (KDIGO) guidelines.^27^ Urine output criteria were not used because urine volume was not available in this study. To minimize the misclassification of non-AKI, we required a maximal gap of 14 days between any 2 consecutive SCr tests. The earliest day that the SCr level change met the KDIGO criteria was established as the date of AKI onset. For patients without detected AKI, their AKI status was considered censored after the last SCr test that met the testing gap requirement. The stage of AKI was identified using the peak SCr level after AKI detection, with an increase of less than 100% being defined as stage 1, 100% or greater as stage 2, and 200% or more over baseline as stage 3.

All drugs prescribed during hospitalization were classified according to the Anatomical Therapeutic Chemical classification system. Exposure to ibuprofen was coded as a time-dependent dichotomous variable.^28^ The cumulative dose of the drug within the exposure period was calculated and standardized by the body weight of the child. Concomitant use of other drugs with nephrotoxic effects (eAppendix 1 in the Supplement) was defined similarly.

The presence of comorbidities was defined by dichotomous covariates according to *ICD-10-CM* diagnosis codes at admission and discharge (eAppendix 2 in the Supplement). The comorbidities analyzed included fever, trauma, tumors, sepsis, epilepsy, heart failure, liver disease, lung infection, respiratory infection, chronic kidney disease (CKD), diarrhea, congenital heart disease, anemia, brain injury, and intracranial infection. The clinical procedures were classified into gastrointestinal, cardiothoracic, neurosurgical, orthopedic, respiratory, urinary, and other operations according to *ICD-10-CM* codes.

### Statistical Analysis

Continuous variables were presented as a mean (SD) or a median (interquartile range [IQR]), whereas categorical data were presented as a number (percentage). An unpaired, 2-tailed *t* test or a Wilcoxon test was used to test group differences for continuous variables, and the Pearson χ^2^ test was used for categorical variables. The association between ibuprofen and the risk of hospital-acquired AKI was examined using a Cox proportional hazards regression model after adjusting for prespecified covariates, including age, sex, standardized baseline SCr level, comorbidities (fever, trauma, tumors, sepsis, epilepsy, heart failure, liver disease, lung infection, respiratory infection, CKD, diarrhea or vomiting, congenital heart disease, anemia, brain injury, and intracranial infection), clinical procedures (gastrointestinal, cardiothoracic, neurosurgical, orthopedic, respiratory, urinary, intervention, and other operations), and concomitant use of other drugs with nephrotoxic effects (other NSAIDs, angiotensin-converting enzyme inhibitors or angiotensin receptor blockers, thiazide diuretics, loop diuretics, aminoglycosides, antiepilepsy drugs, antimycotics, contrasts, chemotherapy drugs, proton pump inhibitors, and other antibiotics). Cox proportional hazards regression was performed with stratification by hospital and hospital division and with drug exposure, clinical procedures, and need for intensive care coded as time-dependent variables.

The association of ibuprofen with the risk of hospital-acquired AKI was also examined among subgroups that were stratified by age (1 month to 1 year, >1 to 10 years, or >10 to 18 years), sex (male or female), presence of CKD, need for intensive care, and concomitant use of other drugs with nephrotoxic effects. An interaction term was added to the Cox proportional hazards regression model to test for possible effect modification by the grouping factor. A penalized smoothing spline was used in the Cox proportional hazards regression model to obtain the curve of cumulative dose of ibuprofen and the risk of hospital-acquired AKI.

Two sensitivity analyses were performed. In the first sensitivity analysis, the association between use of ibuprofen and risk of hospital-acquired AKI was analyzed in 1:1 propensity score–matched pairs of ibuprofen users and nonusers. The propensity score of ibuprofen use was estimated from a logistic regression model whose covariates included age, sex, hospital division, need for intensive care, baseline SCr level, fever, any tumor, trauma, fracture, brain injury, sepsis, heart failure, CKD, congenital heart disease, liver disease, lung infection, epilepsy, anemia, intracranial infection, dehydration, diarrhea or vomiting, clinical procedure, and use of other drugs with nephrotoxic effects. The following 3 propensity score matching methods were compared: (1) nearest-neighbor matching without replacement and within a caliper width of 0.001; (2) nearest-neighbor matching without replacement and within a caliper width of 0.2 × SD of the logit of the estimated propensity score; and (3) exact matching, according to sex, need for intensive care, and exposure to drugs with nephrotoxic effects, and nearest-neighbor matching without replacement and within a specified caliper width of 0.2 × SD of the logit of the estimated propensity score for other covariates. The value of standardized differences less than 0.10 was considered a satisfactory balance between the 2 groups.^29^

The second sensitivity analysis was performed using pROCK (pediatric reference change value optimized for AKI in children),^30^ a new criterion for pediatric AKI based on the reference change value of SCr. According to pROCK, AKI was an increase in SCr level of both 20 μmol/L and 30% or more over the baseline. A previous study showed that the pROCK criterion was more optimized than the KDIGO criteria in detecting true AKI in children.^30^

All analyses were performed using R, version 3.5.3 (R Foundation for Statistical Computing). A 2-tailed *P* < .05 was considered to be evidence of statistical significance. Data analysis was conducted from January 1, 2020, to August 30, 2020.

## Results

### Study Population

Among 939 976 hospitalized children, 50 420 children met the inclusion and 889 556 met the exclusion criteria (Figure 1). The characteristics of these included and excluded children with prescriptions and who underwent a certain number of SCr tests are summarized in eTable 1 in the Supplement. The distribution of age and sex was similar between the included and excluded groups. Of the 50 420 children selected for analysis (of whom 30 640 were boys [60.8%]), the mean (SD) age was 5.0 (5.2) years, the median (IQR) LOS was 15 (10-21) days, the number of patients who were prescribed ibuprofen during hospitalization was 5526 (11.0%), and the number of patients who developed hospital-acquired AKI was 3476 (6.9%). Children who were excluded from the data set (n = 274 487) tended to have fewer SCr tests, fewer comorbidities, and shorter LOS and were less likely to be ibuprofen users. A total of 13 511 children (26.8%) received at least 1 form of NSAIDs during hospitalization, among which ibuprofen was the most commonly prescribed (eTable 2 in the Supplement).

The demographic and clinical characteristics of the children in the study stratified by ibuprofen use are summarized in Table 1. Compared with nonusers, ibuprofen users tended to be younger (mean [SD] age, 5.1 [5.3] years vs 4.2 [3.9] years) and to have a higher baseline SCr level (median [IQR], 29 [21-41] μmol/L vs 31 [23-42] μmol/L); a longer LOS (median [IQR], 14 [10-21] days vs 18 [12-29] days); a higher rate of respiratory infections (13 309 [29.7%] vs 2262 [40.9%]), tumors (4783 [10.7%] vs 857 [15.5%]), and sepsis (2295 [5.1%] vs 734 [13.3%]); a greater need for intensive care (4373 [9.7%] vs 851 [15.4%]); and a higher rate of in-hospital mortality (288 [0.6%] vs 134 [2.4%]). Diuretics and proton pump inhibitors were the most frequent concomitant drugs prescribed. Among the 5526 ibuprofen users, 427 hospital-acquired AKI events (7.7%) were detected, with a median (IQR) time to event of 9 (6-14) days. The characteristics of ibuprofen users stratified by AKI status are compared in eTable 3 in the Supplement.

**Table 1.** Characteristics of Ibuprofen Users vs Nonusers Among Hospitalized Children

Variable	No. (%)		*P* value^a^
	Ibuprofen user (n = 5526)	Nonuser (n = 44 894)	
Age, mean (SD), y	4.2 (3.9)	5.1 (5.3)	<.001
Male sex	3289 (59.5)	27 351 (60.9)	.04
Baseline SCr, median (IQR), μmol/L	31 (23-42)	29 (21-41)	<.001
Need for intensive care	851 (15.4)	4373 (9.7)	<.001
AKI incidence	427 (7.7)	3049 (6.8)	.009
AKI stage			<.001
1	268 (4.9)	2032 (4.5)	
2	92 (1.7)	724 (1.6)	
3	67 (1.2)	293 (0.7)	
In-hospital death	134 (2.4)	288 (0.6)	<.001
LOS, median (IQR), d	18 (12-29)	14 (10-21)	<.001
PCCCs	2306 (41.7)	16 265 (36.2)	<.001
Comorbidities			
Indications for ibuprofen use			
Fever	397 (7.2)	1186 (2.6)	<.001
Trauma	206 (3.7)	1712 (3.8)	.77
Tumor	857 (15.5)	4783 (10.7)	<.001
CKD	167 (3.0)	2734 (6.1)	<.001
Sepsis	734 (13.3)	2295 (5.1)	<.001
Epilepsy	148 (2.7)	1258 (2.8)	.61
Heart failure	199 (3.6)	1230 (2.7)	.002
Liver disease	117 (2.1)	1702 (3.8)	<.001
Lung infection	1709 (30.9)	8389 (18.7)	<.001
Respiratory infection	2262 (40.9)	13 309 (29.7)	<.001
Diarrhea	101 (1.8)	911 (2.0)	.34
Congenital heart disease	867 (15.7)	7594 (16.9)	.02
Anemia	319 (5.8)	1821 (4.1)	<.001
Brain injury	110 (2.0)	1130 (2.5)	.02
Intracranial infection	494 (8.9)	1794 (4.0)	<.001
Combination therapy			
ACEIs/ARBs	297 (5.4)	1437 (3.2)	<.001
Thiazide diuretics	433 (7.8)	2270 (5.1)	<.001
Loop diuretics	2630 (47.6)	13 123 (29.2)	<.001
Aminoglycosides	195 (3.5)	554 (1.2)	<.001
Antiepilepsy drugs	1119 (20.3)	4726 (10.5)	<.001
Antimycotics	631 (11.4)	1919 (4.3)	<.001
Contrasts	619 (11.2)	1863 (4.2)	<.001
Chemotherapy agents	776 (14.0)	2199 (4.9)	<.001
PPIs	1640 (29.7)	12 735 (28.4)	.04
Clinical procedures			
Gastrointestinal	189 (3.4)	3226 (7.2)	<.001
Cardiothoracic	545 (9.9)	5091 (11.3)	.001
Neurosurgical	96 (1.7)	1204 (2.7)	<.001
Orthopedic	217 (3.9)	1648 (3.7)	.34
Respiratory	220 (4.0)	552 (1.2)	<.001
Urinary	55 (1.0)	890 (2.0)	<.001
Other	48 (0.9)	648 (1.4)	.003

Abbreviations: ACEI, angiotensin-converting enzyme inhibitor; AKI, acute kidney injury; ARB, angiotensin receptor blocker; CKD, chronic kidney disease; IQR, interquartile range; LOS, length of stay; PCCC, pediatric complex chronic condition; PPI, proton pump inhibitor; SCr, serum creatinine.

^a^ *P* values were from tests for differences between ibuprofen users and nonusers.

### Risk of Hospital-Acquired AKI Among Ibuprofen Users

Ibuprofen use was associated with a significantly increased risk of hospital-acquired AKI (hazard ratio [HR], 1.23; 95% CI, 1.14-1.34) after adjusting for confounders (Table 2). The results of association analyses in subgroups are summarized in Table 3. A significantly larger HR for ibuprofen was observed in children of older age (>10 years; adjusted HR, 1.64; 95% CI, 1.32-2.05), with CKD (adjusted HR, 2.31; 95% CI, 1.73-3.10), and who required intensive care (adjusted HR, 1.47; 95% CI, 1.24-1.75).

**Table 2.** Use of Ibuprofen and the Risk of Hospital-Acquired Acute Kidney Injury (AKI) in Hospitalized Children

Group	Total, No.	AKI, No. (%)	Crude HR (95% CI)	*P* value	Adjusted HR (95% CI)^a^	*P* value
Nonuser	44 894	3049 (6.8)	1 [Reference]	NA	1 [Reference]	NA
Ibuprofen user	5526	427 (7.7)	1.36 (1.27-1.46)	<.001	1.23 (1.14-1.34)	<.001

Abbreviations: HR, hazard ratio; NA, not applicable.

^a^ Estimated from a Cox proportional hazards regression model using Kidney Disease Improving Global Outcomes criteria and adjusted for age, sex, standardized baseline creatinine level, pediatric complex chronic conditions, comorbidities, clinical procedures, and use of other nephrotoxic drugs use and stratified by hospital and hospital division (detailed adjustment variables are listed in the Methods section).

**Table 3.** Association Between Ibuprofen Use and Hospital-Acquired Acute Kidney Injury (AKI) in Subgroups of Hospitalized Children

Variable	No. of AKI/No. of patients (%)		Crude HR (95% CI)	*P* value for interaction	Adjusted HR (95% CI)^a^	*P* value for interaction
	Ibuprofen user	Nonuser				
Sex				.61		.45
Male	263/3289 (8.0)	1846/27 351 (6.7)	1.11 (1.01-1.23)		1.26 (1.14-1.40)	
Female	164/2237 (7.3)	1203/17 543 (6.9)	1.07 (0.95-1.20)		1.18 (1.05-1.33)	
Age				<.001		<.001
1 mo to 1 y	136/1260 (10.8)	1532/14 292 (10.7)	0.91 (0.80-1.04)		0.99 (0.86-1.13)	
>1 to 10 y	249/3657 (6.8)	1221/21 537 (5.7)	1.36 (1.23-1.51)		1.36 (1.23-1.52)	
>10 to 18 y	42/609 (6.9)	296/9065 (3.3)	2.05 (1.65-2.55)		1.64 (1.32-2.05)	
CKD				<.001		<.001
No	396/5359 (7.4)	2820/42 160 (6.7)	1.06 (0.98-1.15)		1.19 (1.09-1.29)	
Yes	31/167 (18.6)	229/2734 (8.4)	2.23 (1.66-2.99)		2.31 (1.73-3.10)	
Tumor				.88		.20
No	388/4684 (8.3)	2675/39 832 (6.7)	1.09 (1.00-1.19)		1.26 (1.15-1.38)	
Yes	93/914 (10.2)	372/4673 (8.0)	1.11 (0.93-1.33)		1.11 (0.92-1.34)	
ICU				.22		.03
No	323/4675 (6.9)	2696/40 521 (6.7)	1.06 (0.97-1.14)		1.18 (1.07-1.29)	
Yes	104/851 (12.2)	353/4373 (8.1)	1.19 (1.01-1.41)		1.47 (1.24-1.75)	
ACEIs/ARBs				.45		.58
No	390/5229 (7.5)	2902/43 457 (6.7)	1.09 (1.01-1.19)		1.23 (1.13-1.33)	
Yes	37/297 (12.5)	147/1437 (10.2)	1.23 (0.92-1.64)		1.33 (0.99-1.79)	
Contrast media				.50		.32
No	367/4907 (7.5)	2914/43 031 (6.8)	1.10 (1.01-1.19)		1.25 (1.14-1.36)	
Yes	60/619 (9.7)	135/1863 (7.2)	1.01 (0.81-1.27)		1.10 (0.87-1.39)	
Thiazide diuretics				.06		.09
No	395/5093 (7.8)	2796/42 624 (6.6)	1.13 (1.05-1.23)		1.26 (1.15-1.37)	
Yes	32/433 (7.4)	253/2270 (11.1)	0.85 (0.64-1.14)		0.97 (0.72-1.29)	
Loop diuretics				.33		.57
No	136/2896 (4.7)	1746/31 771 (5.5)	1.02 (0.91-1.14)		1.20 (1.07-1.35)	
Yes	291/2630 (11.1)	1303/13 123 (9.9)	1.10 (0.99-1.22)		1.26 (1.13-1.40)	
PPIs				.01		.39
No	301/3886 (7.7)	2326/32 159 (7.2)	1.05 (0.96-1.15)		1.21 (1.10-1.33)	
Yes	126/1640 (7.7)	723/12 735 (5.8)	1.32 (1.13-1.54)		1.31 (1.12-1.53)	
Other NSAIDs				.25		.17
No	269/3235 (8.3)	2660/36 950 (7.2)	0.80 (0.74-0.86)		1.19 (1.08-1.31)	
Yes	158/2291 (7.0)	389/7944 (5.0)	0.87 (0.76-1.02)		1.34 (1.16-1.55)	

Abbreviations: ACEI, angiotensin-converting enzyme inhibitor; ARB, angiotensin receptor blocker; CKD, chronic kidney disease; HR, hazard ratio; ICU, intensive care unit; NSAID, nonsteroidal anti-inflammatory drug; PPI, proton pump inhibitor.

^a^ Adjusted for age, sex, standardized baseline creatinine level, pediatric complex chronic conditions, comorbidities, clinical procedures, and use of other nephrotoxic drugs and stratified by hospital and division (detailed adjustment variables are listed in the Methods section). An interaction term was added to the model when testing for interaction.

**Figure 2.**
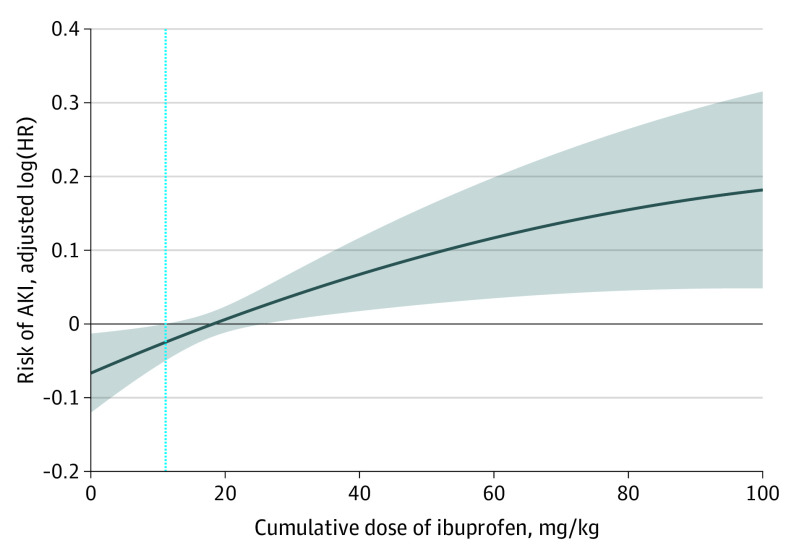
Dose-Response Curve of the Risk of Hospital-Acquired Acute Kidney Injury (AKI) and the Cumulative Dose of Ibuprofen Hazard ratio (HR) was adjusted for covariates in the main text. The dotted vertical line indicates the median cumulative dose of ibuprofen.

Among ibuprofen users, the median (IQR) cumulative dose of ibuprofen was 12.13 (5.59-32.36) mg/kg of body weight. A histogram of the cumulative dose is presented in the eFigure in the Supplement. Children with AKI tended to have a higher cumulative dose of ibuprofen, with a median (IQR) cumulative dose of 13.45 (6.02-44.10) mg/kg in children with AKI and 12.02 (5.55-31.15) mg/kg in children without AKI. The dose-response curve indicates a positive, linear association between ibuprofen exposure and the risk of AKI (Figure 2).

### Sensitivity Analyses

In the first sensitivity analysis, all 3 propensity score matching methods generated well-balanced pairs of ibuprofen users and nonusers, with values of absolute standardized mean differences less than 0.1 (maximal value of 0.07) for all of the covariates considered (eTables 4-6 in the Supplement). The second matching method, which generated the largest number of matched pairs, was used in the subsequent propensity score–matched analysis. Among 5240 propensity score–matched pairs, use of ibuprofen was associated with a significantly increased risk of hospital-acquired AKI (eTable 7 in the Supplement).

In the second sensitivity analysis, we used the pROCK criterion^30^ for detecting hospital-acquired AKI, which has been shown to be more accurate than KDIGO criteria in identifying true AKI. In this analysis, an even larger effect size of ibuprofen on hospital-acquired AKI was observed (HR, 1.38; 95% CI, 1.24-1.54) (eTable 8 in the Supplement).

## Discussion

This large, multicenter, retrospective cohort study revealed that ibuprofen use was common in hospitalized children in China and was associated with a significantly increased risk of hospital-acquired AKI after adjusting for confounders. The association appeared to be dose-dependent. To our knowledge, this study was the first large cohort study to focus on ibuprofen-associated hospital-acquired AKI in Chinese children.

The incidence of hospital-acquired AKI during hospitalization was 6.9% among the 50 420 children included in this study, which was lower than the incidence reported in previous reports.^2,7,9,10^ This discrepancy may be associated with the differences in the characteristics of the study populations, such as the LOS, the disease severity, the frequency of SCr testing, and the method of AKI detection. For example, the study population was drawn from all hospitalized children, and the incidence of hospital-acquired AKI was expected to be lower than the rates in pediatric intensive care.^2^ In addition, we excluded community-acquired AKI (including AKI that occurred in the first 3 days of hospitalization) and did not have urine volume output data, and thus we may have underestimated the incidence of AKI.

In the current study, we found that ibuprofen use was associated with a 23% increased risk of pediatric hospital-acquired AKI after adjusting for confounders (HR, 1.23). This association was robustly observed in the propensity score–matched analysis as well as in the analysis using the pROCK criterion for detecting pediatric AKI. In this study, the estimated HR of ibuprofen for pROCK-defined AKI was larger than the estimated HR for KDIGO-defined AKI (1.38 vs 1.23). This finding was consistent with previous findings that KDIGO criteria were more prone to classifying false AKI as true AKI in children,^30^ thus diluting the estimated effect of ibuprofen use.

A limited number of epidemiological cohort studies have examined the association between ibuprofen use and the risk of pediatric AKI. A case-control study that prospectively enrolled 105 children with acute gastroenteritis found that ibuprofen use was associated with a significantly increased risk of AKI even after adjusting for the degree of dehydration (odds ratio [OR], 2.47; 95% CI, 1.78-3.42).^19^ Balestracci et al^19^ used the pediatric RIFLE criteria as the definition of AKI, and 34 of the 63 patients who received ibuprofen developed AKI. Acute kidney injury occurred more commonly in those who were exposed to 3 or more nephrotoxins (62.5% vs 50.8%), especially in ibuprofen use, although these occurrences were not statistically significant after adjusting for confounders.^20^ In addition, several published case reports found that children sustained AKI after treatment with ibuprofen.^21,22^ A randomized clinical trial among adult ultramarathon runners found that the incidence of AKI was higher among runners who ingested a mean dose of 1200 mg ibuprofen over a 50-mile running course than those who ingested a placebo and that the severity of AKI was worse in the ibuprofen group than in the placebo group.^31^ However, that clinical trial used estimated baseline serum values rather than actual creatinine values, which may make its conclusion unreliable. In contrast, Lahiri et al^23^ showed that high-dose ibuprofen, compared with non–high-dose ibuprofen, was not associated with increased kidney injury molecule-1, N-acetyl-β-glucosaminidase, or urinary protein levels in 52 children with cystic fibrosis. However, the risk of AKI in children who are receiving high-dose ibuprofen and aminoglycoside in the context of volume depletion has been previously described.^24,25^

Within the study population, ibuprofen use was associated with a greater hazard in children who had CKD vs those without (HR, 2.31 [95% CI, 1.73-3.10] vs 1.19 [95% CI, 1.09-1.29]), required intensive care vs those who did not (HR, 1.47 [95% CI, 1.24-1.75] vs 1.18 [95% CI, 1.07-1.29]), or were older vs younger (>10 years or >1 to 10 years vs 1 month to 1 year) (HR, 1.64 [95%CI, 1.32-2.05]; HR, 1.36 [95% CI, 1.23-1.51] vs 0.99 [95% CI, 0.86-1.13]). The exact mechanisms for these interactions were not clear. Children with CKD may have an elevated in vivo drug concentration, and children with more risk factors for developing AKI may be more susceptible to the potential nephrotoxic effect of ibuprofen. A study has suggested that use of ibuprofen in patients with more comorbidities was more likely to be associated with AKI.^32^ In another report, the combined use of ibuprofen and acetaminophen in children was associated with a significantly increased risk of AKI compared with use of these 2 drugs alone.^33^ However, we did not observe a significant interaction of ibuprofen use with use of other classes of drugs, including angiotensin-converting enzyme inhibitors and angiotensin-II receptor blockers, diuretics, proton pump inhibitors, other NSAIDs, and contrast media. The judicious use of ibuprofen and close monitoring of kidney function in children are needed.

### Strengths and Limitations 

This study has several strengths. A major strength is that it used complete and reliable data from a multicenter, large-sample cohort study. Another key strength is that AKI was diagnosed according to a change in SCr level rather than the *ICD-10-CM* code given that basing a diagnosis on *ICD-10-CM* code tends to produce more false-negative diagnoses.

This study has several limitations. First, we did not have urine output levels for this cohort, preventing us from identifying AKI using urine output data. Lack of such data may have led to underestimation of the incidence of AKI. Second, we did not have long-term follow-up data for the children, preventing us from exploring the impact of long-term use of ibuprofen. Third, the cohort consisted of Chinese children only. Whether the findings are generalizable to other ethnic populations requires further examination. Fourth, as in all other association studies, this study may still have unknown confounders, and causal inference is not possible.

## Conclusions

Ibuprofen was widely used and associated with a significantly increased risk of hospital-acquired AKI in hospitalized children in China, particularly children with CKD, of older age, or in need of intensive care. The findings called for judicious use of ibuprofen and close monitoring of kidney function in children.

## Notes

Supplement. **eTable 1.** Characteristics of Hospitalized Children in the Included and Excluded Dataset **eTable 2.** Proportion of NSAIDs in Hospitalized Children **eTable 3.** Characteristics of Hospitalized Children Stratified by Patients With Ibuprofen-Associated AKI Versus Those Treated With Ibuprofen Without AKI **eTable 4.** Characteristics of 1:1 Propensity Score-Matched Cohorts of Ibuprofen Users and Non-Users (Model 1) **eTable 5.** Characteristics of 1:1 Propensity Score-Matched Cohorts of Ibuprofen Users and Non-Users (Model 2) **eTable 6.** Characteristics of 1:1 Propensity Score-Matched Cohorts of Ibuprofen Users and Non-Users (Model 3) **eTable 7.** Use of Ibuprofen and the Risk of HA-AKI in 1:1 Propensity Score-Matched Cohorts by Cox Proportional Hazard Using KDIGO Criteria **eTable 8.** Use of Ibuprofen and the Risk of HA-AKI in Patients by Cox Proportional Hazard Using pROCK Criteria **eFigure.** Histogram of Cumulative Drug Usage of Ibuprofen **eAppendix 1.** Analyzed Nephrotoxic Drugs **eAppendix 2.** Diagnosis Codes (ICD-10)
