# Supplementary material for: Association of Ibuprofen Prescription With Acute Kidney Injury Among Hospitalized Children in China
Source: JAMA Netw Open. 2021 Mar 4;4(3):e210775. doi: 10.1001/jamanetworkopen.2021.0775 (PMC7933997; doi:10.1001/jamanetworkopen.2021.0775)
Supplement: Supplement. — eTable 1. Characteristics of Hospitalized Children in the Included and Excluded Dataset eTable 2. Proportion of NSAIDs in Hospitalized Children eTable 3. Characteristics of Hospitalized Children Stratified by Patients With Ibuprofen-Associated AKI Versus Those Treated With Ibuprofen Without AKI eTable 4. Characteristics of 1:1 Propensity Score-Matched Cohorts of Ibuprofen Users and Non-Users (Model 1) eTable 5. Characteristics of 1:1 Propensity Score-Matched Cohorts of Ibuprofen Users and Non-Users (Model 2) eTable 6. Characteristics of 1:1 Propensity Score-Matched Cohorts of Ibuprofen Users and Non-Users (Model 3) eTable 7. Use of Ibuprofen and the Risk of HA-AKI in 1:1 Propensity Score-Matched Cohorts by Cox Proportional Hazard Using KDIGO Criteria eTable 8. Use of Ibuprofen and the Risk of HA-AKI in Patients by Cox Proportional Hazard Using pROCK Criteria eFigure. Histogram of Cumulative Drug Usage of Ibuprofen eAppendix 1. Analyzed Nephrotoxic Drugs eAppendix 2. Diagnosis Codes (ICD-10) [file jamanetwopen-e210775-s001.pdf]

Supplementary Online Content

Su L, Li Y, Xu R, et al; EACH Study Investigators. Association of ibuprofen prescription with acute kidney injury among hospitalized children in China. *JAMA Netw Open*. 2021;4(3):e210775. doi:10.1001/jamanetworkopen.2021.0775

eTable 1. Characteristics of Hospitalized Children in the Included and Excluded Dataset
eTable 2. Proportion of NSAIDs in Hospitalized Children
eTable 3. Characteristics of Hospitalized Children Stratified by Patients With Ibuprofen-Associated AKI Versus Those Treated With Ibuprofen Without AKI
eTable 4. Characteristics of 1:1 Propensity Score-Matched Cohorts of Ibuprofen Users and Non-Users (Model 1)
eTable 5. Characteristics of 1:1 Propensity Score-Matched Cohorts of Ibuprofen Users and Non-Users (Model 2)
eTable 6. Characteristics of 1:1 Propensity Score-Matched Cohorts of Ibuprofen Users and Non-Users (Model 3)
eTable 7. Use of Ibuprofen and the Risk of HA-AKI in 1:1 Propensity Score-Matched Cohorts by Cox Proportional Hazard Using KDIGO Criteria
eTable 8. Use of Ibuprofen and the Risk of HA-AKI in Patients by Cox Proportional Hazard Using pROCK Criteria
eFigure. Histogram of Cumulative Drug Usage of Ibuprofen
eAppendix 1. Analyzed Nephrotoxic Drugs
eAppendix 2. Diagnosis Codes (ICD-10)

This supplementary material has been provided by the authors to give readers additional information about their work.

©2021 Su L et al. *JAMA Network Open.*

eTable 1. Characteristics of Hospitalized Children in the Included and Excluded Dataset

Variable	Included in the analysis dataset N= 50,420	Excluded from the analysis dataset[#] N= 274,487	P value
Age, mean (SD), year	4.97 (5.16)	5.09 (4.54)	<0.001
Male, n (%)	30,640 (60.77)	176,366 (64.25)	<0.001
Need for intensive care, n (%)	5,224 (10.4)	15,632 (5.69)	<0.001
In-hospital death, n (%)	422 (0.84)	487 (0.18)	<0.001
Length of stays, median (IQR)	15 (10, 21)	7 (4, 11)	<0.001
Daily cost (CNY)	1,292 (757, 2,242)	869 (620, 1304)	<0.001
PCCCs, (%)	18,571 (36.83)	29,384 (10.71)	<0.001
**Comorbidities,** n **(%)**			
Indications for ibuprofen use			
Fever	1,583 (3.14)	8,994 (3.28)	0.13
Trauma	1,918 (3.8)	8,546 (3.11)	<0.001
Tumor	5,640 (11.19)	13,176 (4.8)	<0.001
CKD	2,901 (5.75)	4,100 (1.49)	<0.001
Sepsis	3,029 (6.01)	3,299 (1.2)	<0.001
epilepsy	1,406 (2.79)	9,247 (3.37)	<0.001
Heart failure	1,429 (2.83)	1,489 (0.54)	<0.001
Liver disease	1,819 (3.61)	1,472 (0.54)	<0.001
Lung infection	10,098 (20.03)	33,880 (12.34)	<0.001
Respiratory infection	15,571 (30.88)	63,121 (23)	<0.001
Diarrhea/vomit	1,012 (2.01)	3,255 (1.19)	<0.001
Congenital heart disease	8,461 (16.78)	8,480 (3.09)	<0.001
Anemia	2,140 (4.24)	3,623 (1.32)	<0.001
Brain injury	1,240 (2.46)	4,125 (1.5)	<0.001
Intracranial infection	2,288 (4.54)	7,464 (2.72)	<0.001
**Combination therapy, n (%)**			
ACEI/ARB	1,734 (3.44)	2,004 (0.73)	<0.001
Thiazide diuretic	2,703 (5.36)	1,487 (0.54)	<0.001
Loop diuretic	15,753 (31.24)	12,916 (4.71)	<0.001
Aminoglycoside	749 (1.49)	3,370 (1.23)	<0.001
Anti-epilepsy	5,845 (11.59)	15,607 (5.69)	<0.001
Antimycotics	2,550 (5.06)	1,755 (0.64)	<0.001
Contrast	2,482 (4.92)	7,825 (2.85)	<0.001
Chemotherapy agents	2,975 (5.9)	4,082 (1.49)	<0.001

©2021 Su L et al. *JAMA Network Open.*

Variable	Included in the analysis dataset N=50,420	Excluded from the analysis dataset[#] N= 274,487	P value
PPIs	14,375 (28.51)	28,922 (10.54)	<0.001
Ibuprofen	5,526 (11.0)	14,108 (5.1)	<0.001
Operation, n (%)			
Gastrointestinal operation	3,415 (6.77)	7,993 (2.91)	<0.001
Cardiothoracic operation	5,636 (11.18)	494 (9.69)	<0.001
Neurosurgical operation	1,300 (2.58)	2,517 (0.92)	<0.001
Orthopedic operation	1,865 (3.7)	19,620 (7.15)	<0.001
Respiratory operation	772 (1.53)	6,949 (2.53)	<0.001
Urinary operation	945 (1.87)	3,777 (1.38)	0.16
Other operation	696 (1.38)	15,081 (5.49)	<0.001

Age is presented as mean ± SD. Length of stay and daily cost are presented in median (25th, 75th percentile).

[#]Patients had prescription data but were excluded due to insufficient SCr testing.

Abbreviation: SD, standard deviation; IQR, inter quartile range; CNY, Chinese yuan; PCCCs, pediatric complex chronic conditions; CKD, chronic kidney disease; ACEI, angiotensin converting enzyme inhibitors; ARB, angiotensin receptor blocker; PPIs, proton pump inhibitors.

©2021 Su L et al. *JAMA Network Open.*

eTable 2. Proportion of NSAIDs in Hospitalized Children

NSAIDs	ATC code	N (%)
Ibuprofen	M01AE01	5,526 (11.0)
Acetaminophen	N02BE01	3,691 (7.3)
Acetylsalicylic acid	N02BA01	2,751 (5.5)
Diclofenac	M01AB05	2,566 (5.1)
Flurbiprofen	M01AE09	616 (1.2)
R-ibuprofen	M01AE14	463 (0.9)
Celecoxib	M01AH01	146 (0.3)

Abbreviation: NSAIDs, non-steroid anti-inflammatory drugs; ATC, Anatomical Therapeutic Chemical.

eTable 3. Characteristics of Hospitalized Children Stratified by Patients With Ibuprofen-Associated AKI Versus Those Treated With Ibuprofen Without AKI

Variable	Ibuprofen-associated AKI N= 427	Non-ibuprofen-associated AKI N= 5,099	P value
Age, mean (SD), year	3.64 (3.95)	4.2 (3.88)	0.004
Male, n (%)	263 (61.59)	3026 (59.34)	0.38
Baseline SCr, median (IQR), umol/L	23 (17, 32)	32 (23, 42)	<0.001
Need for intensive care, n (%)	104 (24.36)	747 (14.65)	<0.001
AKI incidence, n (%)	427 (7.73)	-	-
AKI stage, n (%)			-
Stage 1	268 (62.76)	-	-
Stage 2	92 (21.55)	-	-
Stage 3	67 (15.69)	-	-
In-hospital death, n (%)	52 (12.18)	82 (1.61)	<0.001
Length of stays, median (IQR)	22 (15, 35)	18 (12, 29)	<0.001
Daily cost (CNY)	2115 (1345, 3327)	1550 (1003, 2379)	<0.001
PCCCs, (%)	184 (43.09)	2122 (41.62)	0.58
**Comorbidities,** n **(%)**			
Indications for Ibuprofen use			
Fever	18 (4.22)	379 (7.43)	0.01
Trauma	13 (3.04)	193 (3.79)	0.52
Tumor	72 (16.86)	785 (15.4)	0.43
CKD	31 (7.26)	136 (2.67)	<0.001
Sepsis	79 (18.5)	655 (12.85)	0.002
epilepsy	3 (0.7)	145 (2.84)	0.02
Heart failure	30 (7.03)	169 (3.31)	0.001
Liver disease	9 (2.11)	108 (2.12)	1.00
Lung infection	164 (38.41)	1545 (30.3)	0.002
Respiratory infection	194 (45.43)	2068 (40.56)	0.06
Diarrhea/vomit	8 (1.87)	93 (1.82)	1.00
Congenital heart disease	80 (18.74)	787 (15.43)	0.07
Anemia	35 (8.2)	284 (5.57)	0.03
Brain injury	7 (1.64)	103 (2.02)	0.71
Intracranial infection	35 (8.2)	459 (9)	0.60
**Combination therapy, n (%)**			
ACEI/ARB	37 (8.67)	260 (5.1)	0.004

©2021 Su L et al. *JAMA Network Open.*

Thiazide diuretic	32 (7.49)	401 (7.86)	0.84

Variable	Ibuprofen user N=427	Non-Ibuprofen user N= 5,099	P value
Loop diuretic	291 (68.15)	2339 (45.87)	<0.001
Aminoglycoside	29 (6.79)	166 (3.26)	0.002
Anti-epilepsy	116 (27.17)	1003 (19.67)	<0.001
Antimycotics	73 (17.1)	558 (10.94)	<0.001
Contrast	60 (14.05)	559 (10.96)	0.06
Chemotherapy agents	58 (13.58)	718 (14.08)	0.83
PPIs	126 (29.51)	1514 (29.69)	0.96
**Operation, n (%)**			
Gastrointestinal operation	8 (1.87)	181 (3.55)	0.07
Cardiothoracic operation	51 (11.94)	494 (9.69)	0.16
Neurosurgical operation	7 (1.64)	89 (1.75)	1.00
Orthopedic operation	19 (4.45)	198 (3.88)	0.60
Respiratory operation	35 (8.2)	185 (3.63)	<0.001
Urinary operation	4 (0.94)	51 (1)	1.00
Other operation	2 (0.47)	46 (0.9)	0.43

Age is presented as mean ± SD. Baseline SCr, Length of stay and daily cost are presented in median (25th, 75th percentile).

Abbreviation: AKI, acute kidney injury; SD, standard deviation; IQR, inter quartile range; SCr, serum creatinine; CNY, Chinese yuan; PCCCs, pediatric complex chronic conditions; CKD, chronic kidney disease; ACEI, angiotensin converting enzyme inhibitors; ARB, angiotensin receptor blocker; PPIs, proton pump inhibitors.

©2021 Su L et al. *JAMA Network Open.*

eTable 4. Characteristics of 1:1 Propensity Score-Matched Cohorts of Ibuprofen Users and Non-Users (Model 1)

Variable	Ibuprofen user N=4,729	Non-Ibuprofen user N= 4,729	Standardize difference
Age, mean (SD), year	4.04 (3.83)	3.99 (4.56)	0.0128
Male, n (%)	2,839 (60.03)	2,836 (59.97)	0.0013
Baseline SCr, median (IQR), umol/L	31 (22, 33)	30 (21, 34)	-0.0118
Need for intensive care, n (%)	606 (12.81)	624 (13.2)	-0.0105
**Comorbidities, n (%)**			
Indications for Ibuprofen use			
Fever	201 (4.25)	195 (4.12)	0.0049
Trauma	189 (4)	206 (4.36)	-0.0190
Tumor	639 (13.51)	655 (13.85)	-0.0093
CKD	164 (3.47)	166 (3.51)	-0.0025
Sepsis	393 (8.31)	413 (8.73)	-0.0125
epilepsy	116 (2.45)	106 (2.24)	-0.0131
Heart failure	183 (3.87)	186 (3.93)	-0.0034
Liver disease	115 (2.43)	103 (2.18)	0.0176
Lung infection	1,271 (26.88)	1,353 (28.61)	-0.0375
Respiratory infection	1,758 (37.17)	1,790 (37.85)	-0.0138
Diarrhea	87 (1.84)	86 (1.82)	0.0016
Congenital heart disease	855 (18.08)	926 (19.58)	-0.0413
Anemia	243 (5.14)	246 (5.2)	-0.0027
Brain injury	104 (2.2)	118 (2.5)	-0.0212
Intracranial infection	323 (6.83)	311 (6.58)	0.0089
**Combination therapy, n (%)**			
ACEI/ARB	39 (0.82)	30 (0.63)	0.0219
Thiazide diuretic	63 (1.33)	51 (1.08)	0.0239
Loop diuretic	480 (10.15)	467 (9.88)	0.0087
Anti-epilepsy	235 (4.97)	198 (4.19)	0.0337
Antimycotics	26 (0.55)	26 (0.55)	0.0000
Contrast	97 (2.05)	84 (1.78)	0.0192
Chemotherapy agents	35 (0.74)	37 (0.78)	-0.0042
PPI	296 (6.26)	314 (6.64)	-0.0158
**Operation, n (%)**			
Gastrointestinal operation	27 (0.57)	36 (0.76)	-0.0268
Neurosurgical operation	10 (0.21)	14 (0.3)	-0.0168
Orthopedic operation	16 (0.34)	15 (0.32)	0.0031
Respiratory operation	9 (0.19)	9 (0.19)	0.0000

Abbreviation: SD, standard deviation; IQR, inter quartile range; SCr, serum creatinine; CKD, chronic kidney disease; ACEI, angiotensin converting enzyme inhibitors; ARB, angiotensin receptor blocker; PPIs, proton pump inhibitors.

©2021 Su L et al. *JAMA Network Open.*

eTable 5. Characteristics of 1:1 Propensity Score-Matched Cohorts of Ibuprofen Users and Non-Users (Model 2)

Variable	Ibuprofen user N=5,240	Non-Ibuprofen user N= 5,240	Standardize difference
Age, mean (SD), year	4.04 (3.83)	3.99 (4.56)	0.0155
Male, n (%)	2,839 (60.03)	2,836 (59.97)	0.0031
Baseline SCr, median (IQR), umol/L	31 (23, 42)	31 (21, 42)	-0.0115
Need for intensive care, n (%)	769 (14.56)	785 (14.86)	-0.0307
**Comorbidities, n (%)**			
Indications for Ibuprofen use			
Fever	318 (6.02)	301 (5.7)	0.0067
Trauma	203 (3.84)	191 (3.62)	-0.0040
Tumor	803 (15.2)	822 (15.56)	-0.0227
CKD	167 (3.16)	157 (2.97)	0.0067
Sepsis	599 (11.34)	606 (11.47)	-0.0101
epilepsy	131 (2.48)	137 (2.59)	0.0071
Heart failure	192 (3.63)	211 (3.99)	-0.0123
Liver disease	117 (2.22)	105 (1.99)	0.0278
Lung infection	1,547 (29.29)	1,585 (30.01)	-0.0149
Respiratory infection	2,077 (39.32)	2,111 (39.97)	0.0054
Diarrhea	95 (1.8)	91 (1.72)	-0.0157
Congenital heart disease	866 (16.4)	873 (16.53)	-0.0435
Anemia	291 (5.51)	306 (5.79)	0.0025
Brain injury	107 (2.03)	97 (1.84)	-0.0014
Intracranial infection	405 (7.67)	406 (7.69)	-0.0100
**Combination therapy, n (%)**			
ACEI/ARB	41 (0.78)	55 (1.04)	-0.0176
Thiazide diuretic	63 (1.19)	92 (1.74)	0.0126
Loop diuretic	581 (11)	548 (10.37)	-0.0108
Anti-epilepsy	283 (5.36)	275 (5.21)	0.0074
Antimycotics	16 (0.3)	7 (0.13)	-0.013
Contrast	109 (2.06)	93 (1.76)	0.0093
Chemotherapy agents	53 (1)	48 (0.91)	0.0093
PPI	322 (6.1)	395 (7.48)	-0.021
**Operation, n (%)**			
Gastrointestinal operation	27 (0.51)	39 (0.74)	-0.0215
Neurosurgical operation	13 (0.25)	9 (0.17)	-0.0114
Orthopedic operation	26 (0.49)	17 (0.32)	-0.0056
Respiratory operation	55 (1.04)	18 (0.34)	0.0069

Abbreviation: SD, standard deviation; IQR, inter quartile range; SCr, serum creatinine; CKD, chronic kidney disease; ACEI, angiotensin converting enzyme inhibitors; ARB, angiotensin receptor blocker; PPIs, proton pump inhibitors.

©2021 Su L et al. *JAMA Network Open.*

eTable 6. Characteristics of 1:1 Propensity Score-Matched Cohorts of Ibuprofen Users and Non-Users (Model 3)

Variable	Ibuprofen user N=5,087	Non-Ibuprofen user N= 5,087	Standardize difference
Age, mean (SD), year	4.1 (3.85)	4.2 (4.67)	-0.0265
Male, n (%)	3,069 (60.33)	3,069 (60.33)	0.0000
Baseline SCr, median (IQR), umol/L	31 (23, 41)	31 (22, 42)	-0.0157
Need for intensive care, n (%)	671 (13.19)	671 (13.19)	0.0000
**Comorbidities, n (%)**			
Indications for Ibuprofen use			
Fever	280 (5.5)	263 (5.17)	0.0129
Trauma	195 (3.83)	211 (4.15)	-0.0166
Tumor	754 (14.82)	880 (17.3)	-0.0684
CKD	166 (3.26)	174 (3.42)	-0.0092
Sepsis	524 (10.3)	534 (10.5)	-0.0058
epilepsy	122 (2.4)	138 (2.71)	-0.0195
Heart failure	191 (3.75)	210 (4.13)	-0.0200
Liver disease	116 (2.28)	88 (1.73)	0.0382
Lung infection	1,465 (28.8)	1,467 (28.84)	-0.0009
Respiratory infection	3,114 (61.21)	3,157 (62.06)	0.0172
Diarrhea	90 (1.77)	91 (1.79)	-0.0015
Congenital heart disease	858 (16.87)	969 (19.05)	-0.0600
Anemia	280 (5.5)	290 (5.7)	-0.0084
Brain injury	106 (2.08)	113 (2.22)	-0.0099
Intracranial infection	361 (7.1)	362 (7.12)	-0.0007
**Combination therapy, n (%)**			
ACEI/ARB	38 (0.75)	50 (0.98)	-0.0272
Thiazide diuretic	63 (1.24)	69 (1.36)	-0.0111
Loop diuretic	525 (10.32)	605 (11.89)	-0.0496
Anti-epilepsy	247 (4.86)	277 (5.45)	-0.0254
Antimycotics	32 (0.63)	40 (0.79)	-0.0179
Contrast	105 (2.06)	107 (2.1)	-0.0027
Chemotherapy agents	43 (0.85)	57 (1.12)	-0.0275
PPI	310 (6.09)	351 (6.9)	-0.0335
**Operation, n (%)**			
Gastrointestinal operation	28 (0.55)	34 (0.67)	-0.0166
Neurosurgical operation	10 (0.2)	12 (0.24)	-0.0078
Orthopedic operation	20 (0.39)	17 (0.33)	0.0086
Respiratory operation	17 (0.33)	17 (0.33)	0.0000

Abbreviation: SD, standard deviation; IQR, inter quartile range; SCr, serum creatinine; CKD, chronic kidney disease; ACEI, angiotensin converting enzyme inhibitors; ARB, angiotensin receptor blocker; PPIs, proton pump inhibitors.

©2021 Su L et al. *JAMA Network Open.*

eTable 7. Use of Ibuprofen and the Risk of HA-AKI in 1:1 Propensity Score-Matched Cohorts by Cox Proportional Hazard Using KDIGO Criteria

	**Matched-Pairs**	**No. of AKI**	**Adjusted[a] HR (95% CI)**	**P value**
Model1	4,729	713	1.144 (1.027-1.273)	0.014
Model2	5,240	823	1.144 (1.036-1.264)	0.008
Model3	5,087	791	1.143 (1.035-1.264)	0.009

Abbreviation: KDIGO: Kidney Disease Improving Global Outcomes; HR: Hazard Ratio; CI, confidence interval.

Model1: nearest-neighbor matching without replacement and within specified caliper width (0.001);

Model2: nearest-neighbor matching without replacement and within specified caliper width (0.2*sd of the logit of the estimated propensity score);

Model3: exact matching on sex, need for intensive care and use of nephrotoxic drugs and nearest-neighbor matching without replacement and within specified caliper width (0.2*sd of the logit of the estimated propensity score) on other covariates.

a: Adjusted age, sex, standardized baseline creatinine, need for intensive care, PCCCs, comorbidities, clinical procedures, other nephrotoxic drugs use and stratified by hospital and division (Detailed adjustment variables in methods).

©2021 Su L et al. *JAMA Network Open.*

eTable 8. Use of Ibuprofen and the Risk of HA-AKI in Patients by Cox Proportional Hazard Using pROCK Criteria

	**Total, N**	**No. of AKI**	**Crude HR (95% CI)**	**P value**	**Adjusted[a] HR (95% CI)**	**P value**
Non-user	44,894	1,376	Reference		Reference	
Ibuprofen	5,526	242	1.64 (1.49-1.80)	<0.001	1.38 (1.24-1.54)	<0.001

Abbreviation: pROCK: pediatric reference change value optimized for AKI in children; HR: Hazard Ratio.

a: Adjusted age, sex, standardized baseline creatinine, need for intensive care, pediatric complex chronic conditions, comorbidities, clinical procedures, other nephrotoxic drugs use and stratified by hospital and division (Detailed adjustment variables in methods).

©2021 Su L et al. *JAMA Network Open.*

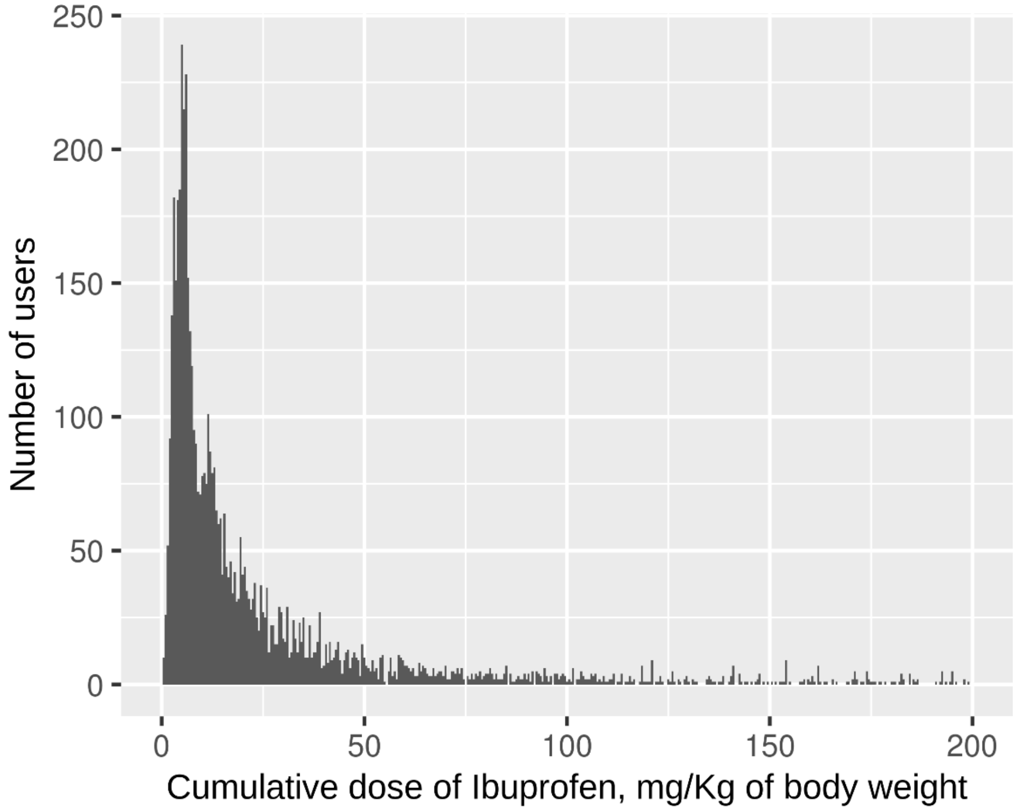

eFigure. Histogram of Cumulative Drug Usage of Ibuprofen

©2021 Su L et al. *JAMA Network Open.*

eAppendix 1. Analyzed Nephrotoxic Drugs

Proton Pump Inhibitors
Omeprazole
Lansoprazole
Pantoprazole
Esomeprazole
Rabeprazole

Aminoglycoside Antibiotic
Gentamicin
Amikacin
Tobramycin
Streptomycin
Etimicin

Antiepileptic Drugs
Sodium Valproate
Phenobarbital
Carbamazepine
Oxcarbazepine
Levetiracetam
Lamotrigine
Topiramate
Clonazepam
Pregabalin
Zonisamide
Gabapentin
Phenytoin Sodium

Antifungal Drugs
Fluconazole
Itraconazole
Voriconazole
Amphotericin B
Caspofungin
Amphotericin
Micafungin Net
Miconazole
Flucytosine
Ketoconazole

©2021 Su L et al. *JAMA Network Open.*

Caspofungin Acetate

Chemotherapeutic Drugs
Vindesine
Daunorubicin
Cisplatin
Cyclophosphamide
Topotecan
Ifosfamide
Carboplatin
Pegaspargase
Cytarabine
Mitoxantrone
Fluorouracil
Doxorubicin
Rituximab
Sirolimus
Idarubicin
Mercaptopurine
Cladribine
Pirarubicin
Pingyangmycin
Doxorubicin
Methotrexate
Epirubicin
Bevacizumab
Paclitaxel
Bleomycin
Dacarbazine
Arsenic Trioxide
Hydroxyurea
Actinomycin D
Nedaplatin
Gemcitabine
Retinoic Acid
Asparaginase
Temozolomide
Oxaliplatin
Tegafur, Gimeracil And Oteracil Porassium
Thioguanine
Ubenimex
Camptothecin
Aclarubicin

©2021 Su L et al. *JAMA Network Open.*

Harringtonine
Mannatide
Calcium Levofolinate
Fludarabine
Brucea Javanica Oil
Docetaxel
Arsenious Acid
Rukuaixiao

Contrast Media
Urografin
Iohexol
Iopamidol
Gadodiamide
Iopromide
Ioversol
Acetone
Iodized Oil
Gadopentetate Dimeglumine
Iodixanol

Nonsteroid Anti-Inflammatory Drug
Ibuprofen
Acetaminophen
Aspirin
Compound Paracetamol And Methylephedrine Oral Solution
Diclofenac
Indomethacin
Compound Pseudoephedrine Hydrochloride
Paracetamol Suppositories
Paracetamol, Pseudoephedrine Hydrochloride,
Dextromethorphan Hydrobromide And Chlorphenamine Maleate Tablets
Sodium Salicylate
Pediatric Paracetamol, Atificial Cow-Bezoar And Chlorphenamine Maleate
Paracetamol, Caffein, Atificial Cow-Bezoar And Chlorphenamine Maleate
Compound Aminophenazone And Barbital
Glucosamine
Pseudoephedrine Hydrochlorid, Chlorpheniramine Maleate
And Dextromethorphan Hydrobromide
Acetaminophen Oral Solution
Dexibuprofen
Flurbiprofen
Etoricoxib

©2021 Su L et al. *JAMA Network Open.*

Parecoxib Sodium
Acetaminophen Oral Solution
Compound Indomethacin Tincture
Paracetamol Solution
Celecoxib
Propacetamol
Superoxide Dismutase
Meloxicam

Other Antibiotics
Vancomycin
Teicoplanin
Sulfonamide
Trimethoprim and Sulfamethoxazole
Compound Sulfamethoxazole

©2021 Su L et al. *JAMA Network Open.*

eAppendix 2. Diagnosis Codes (ICD-10)

Congenital heart disease/ Cardiac surgery: Q24.9, O99.89, I97.110, I97.120, I97.130, I97.710, I97.790, I97.810, I97.820

Chronic kidney disease (Glomerulonephritis + chronic renal insufficiency): N00.2, N00.3, N00.4, N00.5, N00.7, N01.2, N01.4, N01.5, N01.7, N02.2, N02.3, N02.4, N02.5, N02.7, N03.2, N03.3, N03.4, N03.5, N03.7, N04.2, N04.3, N04.4, N04.5, N04.7, N05.2, N05.3, N05.4, N05.5, N05.7, N06.2, N06.3, N06.4, N06.5, N06.7, N07.2, N07.3, N07.4, N07.5, N07.7, N18

Respiratory failure: J95.821, J95.822, J96.00, J96.01, J96.02, J96.10, J96.11, J96.12, J96.20, J96.21, J96.22, J96.90, J96.91, J96.92, P28.5

Shock: A48.3, O03.31, O03.81, O04.81, O07.31, O08.3, O75.1, R45.7, R57.0, R57.1, R57.8, R57.9, R65.21, T75.01, T78.2, T79.4, T81.1, T81.11, T81.12, T81.19, T88.2, Y63.4, Y84.3

Heart failure: I09.81, I11.0, I13.0, I13.2, I50.2, I50.3, I50.4, I50.8, I50.9, I97.13

Urinary tract obstruction: N13.0, N13.2, N32.0, N13.5, N13.8, N13.9

Diabetes: E08.00, E08.01, E08.1, E08.2, E08.3, E08.4, E08.5, E08.6, E08.8, E08.9, E09.0, E09.1, E09.2, E09.3, E09.4, E09.5, E09.6, E09.8, E09.9, E10.1, E10.2, E10.3, E10.4, E10.5, E10.6, E10.8, E10.9, E11.0, E11.1, E11.2, E11.3

Diarrhea: K58.0, K59.1, P78.3, R19.7

Dehydration: P74.1, T67.3, E86.0

Sepsis: A02.1, A22.7, A26.7, A32.7, A40, A41.0, A41.1, A41.2, A41.3, A41.4, A41.5, A41.8, A41.9, A42.7, A54.86, B37.7, O03.37, O03.87, O04.87, O07.37, O08.82, O85, P36, R65.2

Respiratory infection: J06.9, J22, J44.0, J47.0

Intestinal obstruction: K50.012, K50.112, K50.812, K50.912, K51.012, K51.212, K51.312, K51.412, K51.512, K51.812, K56.5, K56.60, K56.69, K91.3, P76

Trauma: G89.11, G89.21, H05.33, H05.42, H40.3, H61.31, K08.11, K08.41, K08.81, K08.82, M18.3, M87.2, O71.8, O71.9, T79.8, T79.9, Z87.828, Z91.49

©2021 Su L et al. *JAMA Network Open.*

Burn: T20.00, T20.01, T20.02, T20.03, T20.04, T20.05, T20.06, T20.07, T20.09, T20.1, T20.2, T20.3, T21.0, T21.1, T21.2, T21.3, T22.0

Anemia: D46.0, D46.1, D46.2, D46.4, D50, D51, D52, D53, D55, D56, D57.4, D58, D59, D61,D62, D63, D64

Malnutrition: E43, E44, E45, E46, E64.0, O25.1, O25.2, O25.3

Epilepsy: G40.00, G40.01, G40.1, G40.2, G40.3, G40.4, G40.80, G40.90, G40.91, G40.B0, G40.B1, Z82.0

Hypoxic ischemic encephalopathy: P91.60, P91.61, P91.62

Urinary tract infection: N39.0, P39.3

Preterm: O42.01, O42.11, O42.91, O60.1, O60.2, P07.3, P59.0

Hematological malignancy:C90, C91, C92, C93, C94, C95, C81, C82, C83, C84, C85, C86

©2021 Su L et al. *JAMA Network Open.*
